# Clinical Features, Diagnostics, Etiology, and Outcomes of Hospitalized Solid Organ Recipients With Community-Acquired Pneumonia: A Retrospective Cohort Analysis

**DOI:** 10.1016/j.chest.2024.05.005

**Published:** 2024-05-30

**Authors:** Oana Joean, Laura Petra von Eynern, Tobias Welte, Gunilla Einecke, Sabine Dettmer, Jan Fuge, Richard Taubert, Heiner Wedemeyer, Jessica Rademacher

**Affiliations:** a Department of Respiratory Medicine and Infectious Diseases, Hannover Medical School, Hannover, Germany; b Biomedical Research in Endstage and Obstructive Lung Disease, Member of the German Center for Lung Research, Hannover, Germany; c Department of Nephrology and Hypertension, Hannover Medical School, Hannover, Germany; d Department of Nephrology and Rheumatology, University Medical Center Goettingen, Goettingen; e Institute of Diagnostic and Interventional Radiology, Hannover Medical School, Hannover, Germany; f Department of Gastroenterology, Hepatology, Infectious Diseases and Endocrinology, Hannover Medical School, Hannover, Germany; g German Centre for Infection Research (H. W.), HepNet Study-House of the German Liver Foundation, Hannover, Germany

**Keywords:** antibiotic therapy, antimicrobial resistance, cardiovascular event, comorbidity, immunosuppression, mortality, *Pseudomonas aeruginosa*, stewardship, transplantation

## Abstract

**Background:**

Community-acquired pneumonia (CAP) is a leading cause of morbidity and mortality worldwide. Limited evidence is available on the most effective diagnostic approaches, management strategies, and long-term outcomes for CAP in patients who have undergone solid organ transplantation.

**Research Question:**

What is the acute and long-term morbidity and mortality after CAP in organ transplant recipients?

**Study Design and Methods:**

We retrospectively analyzed hospitalizations for CAP in solid organ recipients at the largest German transplant center. The study included patients admitted between January 1, 2010, and May 31, 2021. The reported outcomes are in-hospital and 1-year mortality, risk of cardiovascular events during hospitalization and at 1 year, admission to the ICU, and risk of pneumonia with *Pseudomonas aeruginosa*. Multivariable binary logistic regression using stepwise forward selection was performed to determine predictive factors for pneumonia with *P aeruginosa*.

**Results:**

We analyzed data from 403 hospitalizations of 333 solid organ recipients. In > 60% of cases, patients had multiple comorbidities, with cardiovascular and chronic kidney disease being the most prevalent. More than one-half of the patients required oxygen supplementation after admission. In-hospital mortality (13.2%) and the death rate at 1 year postevent (24.6%) were higher than data reported from immunocompetent patients. We also observed high rates of acute cardiovascular events and events occurring 1 year after admission. Early blood cultures and bronchoscopy in the first 24 h significantly increased the odds of establishing an etiology. In our low-resistance setting, the burden of antimicrobial resistance was driven by bacteria from chronically colonized patients, mostly lung transplant recipients.

**Interpretation:**

This comprehensive analysis highlights the high morbidity associated with CAP after transplantation. It also emphasizes the need for prospective multicenter studies to guide evidence-based practices and improve outcomes for these vulnerable patients.

**Take-home Points**

**Study Question:** What are the acute and long-term morbidity and mortality after community-acquired pneumonia in organ transplant recipients?

**Results:** In this study, most patients had multiple comorbidities, with cardiovascular and chronic kidney disease being the most prevalent. In-hospital mortality (13.2%) and death rate at 1 year postevent (24.6%) were higher than rates in immunocompetent people, and we also observed high rates of acute cardiovascular events and events occurring 1 year after admission (23.3% and 43.2%, respectively).

**Interpretation:** Given the high acute and long-term mortality and morbidity after community-acquired pneumonia, prospective, multicentric studies are necessary to investigate risk factors and develop preventive strategies in this population.

Community-acquired pneumonia (CAP) is one of the leading causes of morbidity and mortality associated with infectious diseases worldwide.^1^ Up to one-third of hospitalized patients with CAP have a concurrent immunosuppressive risk factor.^2^, ^3^, ^4^ Although survival in solid organ transplant recipients has increased, these patients still face a high mortality risk due to infectious diseases.^5^, ^6^, ^7^, ^8^ Because immunosuppression is often an exclusion criterion from prospective studies, there is limited evidence on optimal diagnostic approaches, management, and outcomes. Furthermore, the available studies on CAP in solid organ transplant recipients have focused on events in the postoperative time frame, have included few events, or have not reported on long-term outcomes.^9^, ^10^, ^11^, ^12^ Therefore, the available recommendations for CAP treatment in immunocompromised patients reflect the authors’ clinical experience rather than published evidence.^13^^,^^14^

Aiming to bridge this knowledge gap, we performed an extensive retrospective analysis of clinical features, etiology, diagnostic strategies, and outcomes in a German cohort of solid organ transplant recipients hospitalized for CAP over 10 years.

## Study Design and Methods

### Study Design and Setting

We performed a monocentric retrospective cohort analysis of all hospital stays in adult solid organ transplant recipients with a primary diagnosis of CAP between January 1, 2010, and May 31, 2021. Hannover Medical School, where the study was conducted, is the largest academic transplant center in Germany and one of Europe’s largest lung transplant centers. The study was approved by the local institutional review board (No. 9952_BO_K_2021). Due to the retrospective character of the study and the use of only pseudonymized data, no informed consent was necessary. The research was conducted following national standards and the principles of the Declaration of Helsinki. Data were pseudonymized, and only investigators were allowed password-protected access.

### Enrollment and Data Sources

**Figure 1:**
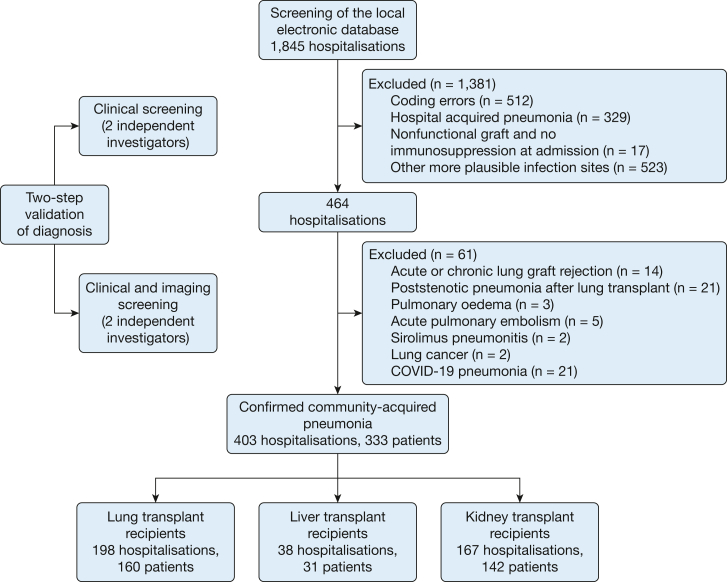
Patient selection flowchart.

Data were collected from the medical records (local transplantation database, hospital stay reports, radiology, laboratory records). Adult (aged ≥ 18 years) patient data were retrieved by using the International Classification of Diseases, 10th revision, codes for pneumonia (J10.0-J18.0). Inclusion required a concomitant code for lung, kidney, or liver transplantation (Z94.0, Z94.2, Z94.3, Z94.4). Inclusion of patients after a combined solid organ transplant was allowed. Combinations of the aforementioned organs and heart or pancreas were also permitted (e-Table 1). Multiple episodes per person were allowed in the analysis. Exclusion criteria were coding errors, pneumonia of noninfectious etiology, pulmonary edema as a primary diagnosis, other infectious diseases as the primary diagnosis, hospital-acquired pneumonia, lung graft rejection, poststenotic pneumonia as a mechanical complication in lung recipients, and COVID-19 pneumonia (Fig 1). Further details on variable definitions and exclusion criteria are available in e-Appendix 1.

Pneumonia was defined as new pulmonary infiltrate or cavitation at the time of admission and at least one of the following criteria: (1) fever; (2) new or increased cough, sputum production, or dyspnea; and (3) signs of systemic inflammation (abnormal WBC count or C-reactive protein or procalcitonin levels).^15^ Two independent investigators retrospectively validated discharge diagnoses.

The follow-up period ended 1 year after the admission, in June 2022, or at the time of death, whichever occurred first. To limit selection bias, all patients who met the abovementioned criteria in the analyzed time frame were included. We regarded bronchial stenosis requiring bronchoscopic desobliteration after lung transplant as a confounder due to the frequent visits to the transplant center and higher risk of hospital-acquired infections. Hence, we excluded these hospitalizations (Fig 1).

### Variables and Outcomes

We collected the following data at the time of hospital admission and during hospital stay: time from transplant, immunosuppression regimen, treatment for acute graft rejection within the previous 6 months (≥ 0.5 mg/kg prednisolone equivalent per day for ≥ 5 days or ≥ 500 mg methylprednisolone equivalent per day for ≥ 3 days), antimicrobial prophylaxis, demographics, comorbidities, antimicrobial regimens and therapy length, pneumonia etiology, diagnostics, chronic bacterial colonization, acute kidney injury, and acute heart failure at admission. Culture-positive tests, sample type, and antibiograms were also gathered. Antibiograms were performed following national or European recommendations at the time of admission.

The analyzed outcomes were the length of hospital stay, in-hospital and 1-year mortality, respiratory insufficiency, admission to the ICU, and risk of pneumonia with *Pseudomonas aeruginosa*. In addition, we report on cardiovascular complications, both during hospitalization and 1 year after the event. To avoid immortal time bias, the analyses concerning mortality were performed on transplant recipients who were only hospitalized once (n = 333). Further details regarding definitions of variables and outcomes are available in e-Appendix 1.

For the analysis, we grouped lung recipients and lung and other organ recipients (eg, liver, kidney, heart) because the immunosuppression level is dictated by the higher levels required to prevent acute lung graft rejection. We hypothesized that the higher immunosuppression level of the patients previously mentioned and the susceptibility to infection of the lung graft are substantial risk factors for CAP. Furthermore, out of the remaining patients, we grouped kidney graft recipients and recipients of a kidney graft and additional organs (eg, pancreas, heart, liver) due to similarities in immunosuppression targets and comorbidities. All analyses were performed on the groups as previously described.

### Statistical Methods

Continuous variables are summarized as median and interquartile range (IQR) (25th-75th percentiles). Categorical variables are reported as number (%). A two-sided *t* test or Mann-Whitney *U* test, χ^2^ test, and analysis of variance test followed by Bonferroni post hoc analysis were used for group comparisons, as appropriate. We performed multivariate binary logistic regression using stepwise forward selection to determine predictive factors for identifying the etiology and pneumonia with *P aeruginosa*. We conducted a complete case analysis. Variable entry was set at *P* < .05 and removal at *P* > .10. Data are reported as ORs and CIs. The Wald method was used for CI calculation.

While building the regression model for the risk of pneumonia with *P aeruginosa,* we observed high collinearity between the variables lung transplantation and previous colonization with *P aeruginosa*. Due to this, we introduced the variables lung transplantation with/without known colonization with *P aeruginosa*. For the other predefined variables, see e-Tables 2 and 3.

All reported *P* values are two-sided, unless indicated otherwise. The data were analyzed using the IBM SPSS Statistics (version 29.0; IBM Corp) program.

## Results

### Participants and Main Outcomes

We included 403 hospitalizations of 333 solid organ recipients (198 stays of lung recipients, 167 kidney graft recipients, and 38 liver transplant recipients), as shown in Figure 1. Most of the patients (96.7%) experienced one or two episodes in the time period analyzed, and only a low number were admitted more frequently. Lung transplant recipients were younger (51 [IQR 33-60] vs 60 [IQR, 53-64] years in liver graft recipients and 59 [IQR, 51-68] years in kidney recipients; *P* < .001). CAP episodes occurred earlier after transplantation in lung transplant recipients than in the other groups (e-Fig 1, Table 1). Most lung graft recipients had an immunosuppression regimen based on prednisolone, calcineurin inhibitors, and mycophenolate mofetil. Furthermore, there were significantly more hospitalizations for CAP in this group after an episode of acute graft rejection, with 71 cases (35.9%) compared with four cases (10.5%) in liver recipients and 40 cases (24%) in kidney recipients (*P* = .002) (Table 1). Over 60% of hospitalizations occurred in patients with multiple comorbidities, with cardiovascular and chronic kidney disease being the most prevalent. Most CAP stays of kidney graft recipients (> 98%) occurred in patients with these comorbidities. Also, significantly more kidney graft recipients showed signs of an acute kidney injury or acute cardiac failure when admitted with CAP (82% and 31.7%, respectively) (Table 1).

**Table 1:** Patient Characteristics

Variable	All Stays	Lung Transplant Recipients	Liver Transplant Recipients	Kidney Transplant Recipients	*P* Value
No. of CAP episodes	403	198 (49.1)	38 (9.4)	167 (41.5)	< .001
Age, y	57 (43-63)	51 (33-60)	60 (53-64)	59 (51-68)	< .001
Female sex	166 (41.2)	95 (48)	18 (47.4)	167 (31.7)	.005
Time from Tx, mo	46 (14-97)	29 (9-72)	80 (30-125)	68 (24-144)	< .001
Maintenance immunosuppression at the time of CAP					
Prednisolone	388 (96.3)	197 (99.5)	26 (68.4)	165 (98.8)	< .001
CNI	366 (91)	196 (99.5)	34 (89.5)	136 (81.4)	< .001
MMF	305 (75.5)	163 (82.3)	25 (65.8)	117 (70.1)	.008
mTOR	56 (13.9)	17 (7.6)	6 (15.8)	35 (21)	.001
Belatacept	4 (1)	0 (0)	0 (0)	4 (2.4)	NA
Azathioprine	29 (7.2)	10 (5.1)	0 (0)	19 (11.4)	.013
Maintenance prednisolone dose	7.5 (5-7.5)	7.5 (5-7.5)	5 (5-10)	5 (5-7.5)	< .001
Acute rejection 6 mo before CAP	115 (28.5)	71 (35.9)	4 (10.5)	40 (24)	.002
Antimicrobial prophylaxis					
PCP^a^	231 (57.3)	193 (97.5)	1 (2.6)	37 (22.2)	< .001
CMV^b^	66 (16.4)	52 (26.3)	2 (5.3)	12 (7.2)	< .001
*Aspergillus* species^c^	185 (45.9)	183 (92.4)	0 (0)	2 (1.2)	< .001
Comorbidities					
CNS disease	62 (15.4)	29 (14.6)	9 (23.7)	24 (14.4)	> .05
Hepatic insufficiency/chronic liver disease	23 (5.7)	8 (4)	13 (34.2)	2 (1.2)	< .001
COPD^d^	39 (9.7)	10 (5.1)	3 (7.9)	26 (15.6)	.003
Diabetes mellitus^e^	145 (36)	74 (37.4)	11 (28.9)	60 (35.9)	> .05
CKD	330 (81.9)	141 (71.2)	24 (63.2)	165 (98.8)	< .001
Cardiovascular disease	336 (83.4)	138 (69.7)	32 (84.2)	166 (98.4)	< .001
Active neoplastic disease	57 (14.1)	17 (8.6)	13 (34.2)	27 (16.2)	< .001
More than two comorbidities	302 (74.9)	117 (59.1)	25 (65.8)	160 (95.8)	< .001
Signs and symptoms at presentation					
Cough	300 (75)	140 (71.4)	24 (63.2)	136 (81.9)	.01
Purulent expectoration	159 (39.7)	79 (40.3)	12 (31.6)	68 (40.7)	> .05
Crackles or bronchial breathing	217 (54.3)	96 (49)	16 (42.1)	105 (63.3)	.007
Altered mental state	47 (11.7)	14 (7.1)	5 (13.2)	28 (16.8)	.01
Acute kidney failure	253 (62.8)	95 (48)	21 (55.3)	137 (82)	< .001
Acute heart failure	76 (18.8)	19 (9.6)	4 (10.5)	53 (31.7)	< .001
Respiratory rate, breaths/min	21 (17-25)	22 (19-26)	19 (16-23)	20 (16-24)	.001
Heart rate, beats/min	90 (79-103)	95 (81-110)	87 (79-98)	86 (72-100)	< .001
Systolic BP, mm Hg	126 (111-141)	123 (108-136)	123 (101-141)	130 (117-147)	.007
Diastolic BP, mm Hg	70 (62-80)	70 (64-80)	70 (60-77)	72 (61-83)	> .05
Temperature, °C	37.3 (36.7-38.2)	37.3 (36.8-38)	37.5 (36.6-38)	37.4 (36.6-38.5)	> .05
Spo_2_, %	93 (89-96)	92 (89-95)	94 (91-98)	94 (88-96)	> .05
Laboratory findings at admission					
CRP, mg/L	104 (45-191)	106 (40-196)	65 (35-98)	116 (55-228)	.001
Procalcitonin, μg/L	0.6 (0.1-2.4)	0.4 (0.1-1.9)	1 (0.2-2.6)	0.8 (0.2-3)	> .05
Leucocyte count, ×10^3^/μL	8.7 (5.6-13.5)	9.5 (5.6-14.7)	7.3 (5.7-12.6)	8.5 (5.8-13.4)	> .05
Thrombocyte count, ×10^3^/μL	226 (162-310)	253 (190-338)	141 (88-221)	213 (146-281)	< .001
Lymphocyte count, ×10^3^/μL	0.9 (0.5-1.6)	1 (0.6-1.7)	0.7 (0.4-1.4)	0.65 (0.3-1.1)	> .05
Neutrophil count, ×10^3^/μL	6.9 (3.2-11)	7.3 (3.3-11.3)	5.6 (2.1-12)	6.2 (2.9-10)	> .05

Values are median (interquartile range), No. (%), or as otherwise indicated. CAP = community-acquired pneumonia; CKD = chronic kidney disease; CMV = cytomegalovirus; CNI = calcineurin inhibitor; CRP = C-reactive protein; MMF = mycophenolate mofetil; mTOR = mammalian target of rapamycin; NA = not available; PCP = *Pneumocystis jirovecii*; Spo_2_ = oxygen saturation; Tx = transplant.

a PCP prophylaxis was performed in > 99% of cases with trimethoprim/sulfamethoxazole.

b CMV prophylaxis was performed with valganciclovir.

c *Aspergillus* species prophylaxis included itraconazole, posaconazole, or voriconazole.

d Patients with lung transplant were included only in case of single lung transplantation.

e Not including patients with kidney-pancreas transplantation who did not require any diabetes treatment after transplant.

Most of the patients were slightly tachypneic and tachycardic at presentation. Only two-thirds presented with cough and even less with productive sputum and typical auscultatory sounds (Table 1). Most patients were afebrile and only a minority presented with leukocytosis (Table 1).

More than one-half of the patients required oxygen supplementation after admission. In-hospital mortality (13.2% overall) and ICU admission rates were similar across groups. Most of the in-hospital death cases occurred after admission to the ICU. Kidney graft recipients had significantly higher rates of in-hospital and 1-year cardiovascular events (31.1% and 59.1%, respectively) (Table 2).

**Table 2:** Outcomes

Variable	All Stays	Lung Transplant Recipients	Liver Transplant Recipients	Kidney Transplant Recipients	*P* Value
No. of CAP episodes	403	198 (49.1)	38 (9.4)	167 (41.5)	< .001
In-hospital CV events	94 (23.3)	33 (16.7)	9 (23.7)	52 (31.1)	.005
CV events 1 y after admission	144 (43.2)	51 (31.8)	13 (43.3)	84 (59.1)	.01
Length of hospital stay, d	12 (7-21)	11 (7-16)	16 (9-28)	13 (7-23)	.041
Oxygen	247 (61.3)	128 (64.6)	21 (55.3)	98 (58.7)	> .05
NIV	64 (15.9)	32 (16.8)	7 (18.4)	25 (15)	> .05
HFO2	15 (3.7)	5 (2.5)	3 (7.9)	7 (4.2)	> .05
Intubation	62 (15.4)	28 (14.1)	6 (15.8)	28 (16.8)	> .05
ECMO	19 (4.7)	11 (5.6)	2 (5.3)	6 (3.6)	> .05
ICU admission	99 (24.6)	47 (23.7)	12 (31.6)	40 (24)	> .05
ICU mortality^a^	36 (10.8)	18 (11.3)	3 (9.7)	15 (10.6)	> .05
In-hospital mortality^a^	44 (13.2)	21 (13.1)	4 (12.9)	19 (13.4)	> .05
1-y mortality^a^	82 (24.6)	47 (29.3)	6 (18.7)	29 (20.4)	.05

Values are median (interquartile range), No. (%), or as otherwise indicated. CAP = community-acquired pneumonia; CV = cardiovascular; ECMO = extracorporeal membrane oxygenation; HFO2 = high-flow oxygen therapy; NIV = noninvasive ventilation.

a To avoid immortal time bias, the analyses concerning mortality were performed on transplant recipients who were only hospitalized once (n = 333).

Less than 4% of hospital stays had missing data.

### Diagnostic Tests, CAP Etiology, and Burden of Antibiotic Resistance

Blood cultures were analyzed in 69% of hospitalizations, but were drawn within 24 h only in 18.9% of cases (Table 3). Sputum sampling was more frequent in kidney transplant recipients. In contrast, bronchoscopies with BAL were performed significantly more often in patients having received lung transplant (170 [85.9%] vs 17 [44.7%] in liver transplant recipients vs 77 [46.1%] in kidney transplant patients, *P* < .001). Respiratory viral analyses and BAL galactomannan were performed significantly more often in lung graft recipients, which could have been associated with the higher bronchoscopy rate (Table 3). Both blood cultures and BAL samples showed a higher positivity rate if analyzed within the first 24 h of presentation (16% and 87.5%, respectively) (e-Fig 2). No prolonged worsening of gas exchange parameters was documented after bronchoscopy.

**Table 3:** Microbiological Diagnosis

Variable	All stays (N = 403)	Lung Transplant Recipients (n = 198)	Liver Transplant Recipients (n = 38)	Kidney Transplant Recipients (n = 167)	*P* Value
Blood cultures drawn	278 (69)	129 (65.2)	30 (78.9)	119 (71.3)	> .05
Blood cultures drawn within 24 h of presentation	76 (18.9)	47 (23.7)	5 (13.2)	24 (14.4)	.048
Sputum analyzed	126 (31.3)	44 (22.2)	15 (39.5)	67 (40.1)	< .001
BAL performed	264 (65.5)	170 (85.9)	17 (44.7)	77 (46.1)	< .001
BAL performed within 24 h of presentation	110 (27.7)	96 (48.5)	2 (5.3)	12 (7.2)	< .001
Pneumococcal urine antigen performed	144 (35.5)	61 (31)	11 (28.9)	72 (43.1)	.036
*Legionella* species urine antigen performed	155 (38.5)	64 (32.3)	14 (36.8)	77 (46.1)	.026
Respiratory viral analysis	246 (61)	151 (76.3)	23 (60.5)	72 (43.1)	< .001
Serum galactomannan	29 (7.2)	13 (6.6)	3 (7.9)	13 (7.8)	> .05
BAL galactomannan	107 (26.6)	72 (36.4)	6 (15.8)	29 (17.8)	< .001

Values are No. (%) or as otherwise indicated.

An etiological diagnosis was found in 227 of 403 cases (56%) overall but more often in lung transplant recipients (144 of 198; 73%). Logistic regression was used to assess the relationship between the performed diagnostic tests and finding a CAP etiology. In this cohort, the odds of establishing an etiological diagnosis were 2.7 times higher (95% CI, 1.42-5.2) if blood cultures were drawn on the first day of admission and increased 6.23 times (95% CI, 3.37-11.52) if a bronchoscopy with BAL was performed in the first 24 h (e-Table 2).

One-half of the identified etiologies were caused by core respiratory pathogens. Almost all *P aeruginosa* isolates (54 of 60) were identified in lung transplant recipients. A logistic regression exploring risk factors for *P aeruginosa* CAP showed that patients with a history of colonization (in this cohort, patients with cystic fibrosis [CF] bronchiectasis and non-CF bronchiectasis) had significantly higher odds of developing *Pseudomonas* species pneumonia (OR, 45.6; 95% CI, 6.98-122.5) than liver graft or kidney graft recipients. Lung transplant recipients without known colonization had four times higher odds of being diagnosed with *Pseudomonas* species pneumonia (95% CI, 1.36-11.8) than recipients of other organs (e-Table 3).

The burden of antimicrobial resistance was caused by CAP with *P aeruginosa*, methicillin-resistant *Staphylococcus aureus*, and *Burkholderia* species, most in patients with known chronic infection with the respective bacteria (data not shown).

Most fungal infections were caused by *Pneumocystis jirovecii* and *Aspergillus* species, whereas *P jirovecii* occurred only in patients without adequate prophylaxis (either no prophylaxis at all or incorrect doses) (Table 4). Although performing viral polymerase chain reaction or galactomannan testing in the BAL were not statistically relevant parameters for finding the etiology, they were clinically relevant tests (e-Table 2). Patients with respiratory viral infections (respiratory syncytial virus, parainfluenza, human metapneumovirus) were administered antiviral therapies, in contrast to recommendations for immunocompetent patients. In addition, a positive galactomannan together with radiologic signs had substantial therapeutic consequences (data not shown).

**Table 4:** Pathogens Identified

Variable	All	Lung Transplant Recipients	Liver Transplant Recipients	Kidney Transplant Recipients
No. of episodes	403	198	38	167
Episodes with identified pathogen	227 (56.3)	144 (73)	12 (32)	71 (43)
Polymicrobial episodes	32 (7.9)	26 (13.1)	2 (5.2)	4 (2.3)
**Total pathogens identified**	264	171	15	82
**Core respiratory pathogens**	**136 (51.5)**	**87 (50.9)**	**9 (0.6)**	**40 (48.74)**
**Core bacteria**	**82 (31)**	**54 (31.6)**	**5 (33.3)**	**23 (28.04)**
*Streptococcus pneumoniae*	17 (6.4)	10 (5.8)	1 (6.7)	6 (7.3)
*Haemophilus influenzae*	13 (5)	10 (5.8)	0 (0)	3 (3.7)
Methicillin-sensitive *Staphylococcus aureus*	29 (10.9)	17 (9.9)	2 (13.3)	10 (12.2)
Methicillin-resistant *S aureus*	14 (5.3)	12 (7)	0 (0)	2 (2.4)
*Klebsiella* species	7 (2.6)	4 (2.3)	2 (13.3)	1 (1.2)
*Proteus* species	1 (0.4)	0 (0)	0 (0)	1 (1.2)
*Moraxella* species	1 (0.4)	1 (0.6)	0 (0)	0 (0)
**Core viruses**	**54 (20.5)**	**33 (19.3)**	**4 (26.7)**	**17 (20.7)**
Influenza	23 (8.7)	9 (5.2)	4 (26.7)	10 (12.2)
RSV	13 (4.9)	10 (5.8)	0 (0)	3 (3.65)
Parainfluenza	9 (3.4)	8 (4.7)	0 (0)	1 (1.2)
hMPV	9 (3.4)	6 (3.5)	0 (0)	3 (3.65)
**Specific pathogens**	**128 (48.5)**	**84 (49.1)**	**7 (46.7)**	**42 (51.2)**
**Specific bacteria**	**75 (28.4)**	**64 (37.4)**	**1 (6.7)**	**10 (12.2)**
*Pseudomonas aeruginosa*	60 (22.7)	54 (31.6)	0 (0)	6 (7.3)
*Nocardia* species	5 (1.9)	5 (2.9)	0 (0)	0 (0)
*Rhodococcus equi*	3 (1.1)	0 (0)	0 (0)	3 (3.65)
*Mycobacterium tuberculosis*	3 (1.1)	2 (1.1)	1 (6.7)	0 (0)
NTM	2 (0.75)	1 (0.6)	0 (0)	1 (1.2)
*Burkholderia multivorans*	2 (0.75)	2 (1.1)	0 (0)	0 (0)
**Specific viruses**	**4 (1.5)**	**3 (1.7)**	**0 (0)**	**1 (1.2)**
CMV	3 (1.1)	2 (1.1)	0	1 (1.2)
VZV	1 (0.4)	1 (0.6)	0	0
**Fungi**	**49 (18)**	**17 (9.9)**	**5 (30)**	**31 (37.8)**
*Pneumocystis jirovecii*	28 (10.6)	2 (1.2)	4 (26.7)	26 (31.7)
*Aspergillus fumigatus*	18 (6.8)	12 (7)	1 (3.3)	5 (6.1)
Non-*Aspergillus* species molds	3 (1.6)	3 (1.7)	0 (0)	0 (0)

Values are No. (%) or as otherwise indicated. Boldface added to improve readability. CMV = cytomegalovirus; hMPV = human metapneumovirus; NTM = nontuberculous mycobacteria; RSV = respiratory syncytial virus; VZV = varicella zoster virus.

Regarding radiology, 99.3% of patients underwent chest radiographs, whereas around 40% underwent CT scans (data not shown).

### Antiinfective and Immunosuppressive Management

In almost one-third of cases, antibiotic therapies had been started before admission, most often with fluoroquinolones, ß-lactams, or oral cephalosporins (e-Table 4). Antibiotics were used during most hospitalizations (99.5%), with nearly 80% of cases involving *Pseudomonas* species-active substances (Table 5). Unlike the low rate of CAP caused by methicillin-resistant *S aureus*, antibiotics active against this bacterium were prescribed in almost 20% of hospitalizations (Table 5). Furthermore, in one-third of hospitalizations, combination therapies with ß-lactams and macrolides or fluoroquinolones were prescribed (data not shown).

**Table 5:** Antiinfective and Immunomodulatory Therapy After Admission

Variable	All Stays (N = 403)	Lung Transplant Recipients (n = 198)	Liver Transplant Recipients (n = 38)	Kidney Transplant Recipients (n = 167)	*P* Value
Antibiotics after admission	401 (99.5)	196 (98)	38 (100)	167 (100)	> .05
Nephrotoxic antibiotics^a^	78 (19.4)	56 (28.3)	9 (23.7)	13 (7.8)	< .001
*Pseudomonas* species active antibiotics^b^	320 (79.4)	153 (77.3)	30 (78.9)	137 (82)	> .05
MRSA active antibiotics^c^	79 (19.6)	37 (18.7)	12 (31.6)	30 (18)	> .05
Carbapenems	117 (29)	87 (44)	13 (32)	17 (10)	< .001
Fluoroquinolones	95 (23.6)	59 (29.8)	14 (36.8)	22 (13.2)	< .001
Macrolides	86 (21.3)	26 (13.1)	10 (26.3)	50 (30)	< .001
Antimycotics after admission	80 (19.8)	42 (21.2)	10 (26.3)	28 (16.8)	> .05
Antivirals after admission	64 (15.9)	30 (15.2)	3 (7.9)	31 (18.6)	> .05
Immunosuppression reduction after admission	288 (71.5)	150 (75.8)	16 (42.1)	122 (73.1)	< .001

Values are No. (%) or as otherwise indicated. MRSA = methicillin-resistant *Staphylococcus aureus*.

a Aminoglycosides, colistin, teicoplanin, and vancomycin.

b Ceftaroline, ceftazidime, ciprofloxacin, colistin, levofloxacin, piperacillin/tazobactam, and meropenem.

c Ceftobiprole, linezolid, fosfomycin, teicoplanin, and vancomycin.

In > 70% of hospitalizations, the immunosuppressive regimen was reduced during the acute phase of illness. This most often happened in lung and kidney graft recipients (Table 5).

## Discussion

To our knowledge, this is the most extensive report of clinical features, etiology, diagnostics, and outcomes in hospitalized patients with CAP after solid organ transplant. The comorbidity burden, especially cardiovascular and renal, but also the in-hospital mortality, was high in comparison with age-adjusted national reports of immunocompetent patients and previous reports from other transplant cohorts.^10^^,^^11^^,^^16^, ^17^, ^18^ Furthermore, the high rate of incident cardiovascular complications added to the toll of morbidity, most notably in renal transplant recipients (Table 2). Early testing showed higher positive blood culture and respiratory sample rates than previous reports.^2^^,^^19^ Antibiotic-resistant bacteria were most often identified in patients with previous colonization; the remaining core respiratory bacteria reflected the local epidemiology.^20^ Despite that, broad-spectrum antibiotics were frequently prescribed (Table 5).^10^

The risk of infection in transplant recipients is a dynamic entity that changes within the patient by the passing of time.^8^ Infection and acute rejection are situated on a continuum of immune reactions, where the balance can be swiftly tilted in either way. In this cohort, a CAP episode requiring hospitalization was preceded by therapy for acute rejection in 28.5% of cases (Table 1). Furthermore, the immunosuppressive regimen was reduced in 71.5% of cases to support immune reconstitution (Table 5). However, to our knowledge, no prospective comparative studies have examined immunosuppressive management strategies in CAP so far.^21^

The differential diagnosis of CAP in transplanted patients is wide ranging and can include infectious and noninfectious etiologies (Fig 1).^12^^,^^14^ Infectious causes reflect epidemiologic exposure and the net state of immunosuppression: transplant recipients are exposed to the same CAP pathogens as their community but face additional risks due to chronic lung damage or opportunistic infections.^8^ Due to immunosuppression, transplant recipients might have atypical presentation. Fever and leukocytosis were rare; abnormalities in chest auscultation were also scarce in this cohort (Table 1). Therefore, a high index of suspicion for infection together with an understanding of the individual net state of immunosuppression and its potential consequences is necessary.

We found that early analysis of blood cultures and respiratory material, particularly after bronchoscopy, increased the odds of finding an etiology, and we investigated this for the first time in a large cohort of transplant recipients (e-Fig 2, e-Table 2). The positivity rate of blood cultures sampled early during the hospitalization was higher than the rates described in the general population, possibly indicating more frequent invasive infections due to immunosuppression.^19^^,^^22^ Although viral and fungal diagnostics were not significant parameters for establishing an etiological diagnosis in this cohort, positive results had substantial therapeutic consequences. Therefore, we think viral epidemiology and individual immune-associated risks for invasive fungal infection should be considered when evaluating a transplant recipient with CAP. Unfortunately, to our knowledge, no prospective studies evaluating different diagnostic algorithms exist so far.

Retrospective data carry a high risk of bias by indication, in this case, the clinical indication for a certain test. Our data were skewed by results from lung transplant recipients, who underwent bronchoscopies the most, thus introducing a detection bias. Although our results confirm previous analyses of increased diagnostic yield of bronchoscopy in immunosuppressed patients, we did not observe the previously reported high rate of intervention-associated side effects.^23^, ^24^, ^25^

Research on diagnostic approaches in transplant recipients is scarce, with test performance typically defined in immunocompetent populations.^14^ The decision on which patients should be referred for a CT scan rather than a conventional chest radiograph has yet to be investigated. Furthermore, in more recent years, nucleic acid-based tests from respiratory material have been increasingly used in immunosuppressed patients.^26^^,^^27^ None of those aforementioned strategies has been prospectively evaluated for patient-relevant outcomes. Hence, there is a great need for prospective studies for immunosuppressed patients with CAP.

Regarding epidemiology, we found that patients with previous *Pseudomonas* species chronic infection (which in our setting were patients transplanted due to CF bronchiectasis or non-CF bronchiectasis) were at high risk for *Pseudomonas* species pneumonia. A minority of lung transplant recipients without prior chronic infection developed *Pseudomonas* species pneumonia, possibly reflecting the risk of chronic lung damage due to bronchiectasis or chronic allograft dysfunction after lung transplant (e-Table 3). Chronic lung damage and prior colonization have been identified as the main predictors of *Pseudomonas* species pneumonia in immunocompetent patients and could be used to guide empirical antibiotic therapy.^28^ Moreover, the burden of antibiotic resistance was highest in patients with known colonization with resistant organisms, and the remaining core respiratory bacteria reflected local epidemiology.^20^ This should be considered when starting empirical therapy and could be used to tailor stewardship interventions. Nonetheless, a pathogen was found in only one-half of hospitalizations, leaving an epidemiologic black box with unclear implications for management and outcomes.

To our knowledge, this is the first report on the high cardiovascular comorbidity and complication burden in hospitalized patients with CAP after transplant. Although the risk of acute and long-term cardiovascular complications in hospitalized immunocompetent patients with CAP has been described, our understanding of this phenomenon is still limited, and no risk reduction strategies exist.^29^, ^30^, ^31^ A recently published study by Müller-Plathe et al^32^ evaluating risk scores for severe pneumonia in kidney transplant recipients with CAP reported a lower in-hospital mortality rate than in this cohort. However, the analysis excluded patients with signs of acute heart failure at presentation.^32^ This exclusion might indirectly suggest grave implications for mortality in this patient group.

Our study’s limitations are related to its retrospective design: as previously mentioned, we face a high risk of bias by indication regarding diagnostics and therapy. Although the higher rate of *Pseudomonas* species pneumonia in lung transplant recipients might be due to detection bias in a population in which bronchoscopies were often performed, similar data have already been published.^28^ Because we performed a single-center analysis, there is a risk of case-mix bias. There might be significant differences in severity between patients admitted at our center and patients who might have received care at hospitals closest to their homes. This discrepancy could explain the higher mortality rate compared with other transplant cohorts.^11^ On the other hand, others have excluded patients with acute cardiac failure at the time of admission for CAP, thus possibly including a selection bias in their analysis.^32^ Another limitation is that we did not have access to reliable data on immunization status against respiratory infections.

## Interpretation

Our study provides comprehensive data on clinical features, etiology, and yield of microbiological diagnostics from the largest cohort of solid organ transplant recipients hospitalized for CAP published to date. Our research also highlights, for the first time, the comorbidity burden, especially cardiovascular and renal, and the high incidence rate of cardiovascular complications during hospitalization. We also show that CAP etiology reflects the local epidemiology in patients who received transplants. The burden of antimicrobial resistance in our low-resistance setting was driven by bacteria from chronically colonized patients, mostly lung transplant recipients. Conversely, the remaining patients did not have an additional risk of infection with resistant bacteria. These results should guide the selection of empirical therapies and could inform the development of stewardship strategies.

However, multicenter, prospective studies with patient-relevant outcomes are imperative to transition from eminence- to evidence-based medicine for transplant recipients.

## Funding/Support

The authors have reported to *CHEST* that no funding was received for this study.

## Financial/Nonfinancial Disclosures

None declared.

## Figures and Tables

**e-Online Data:**
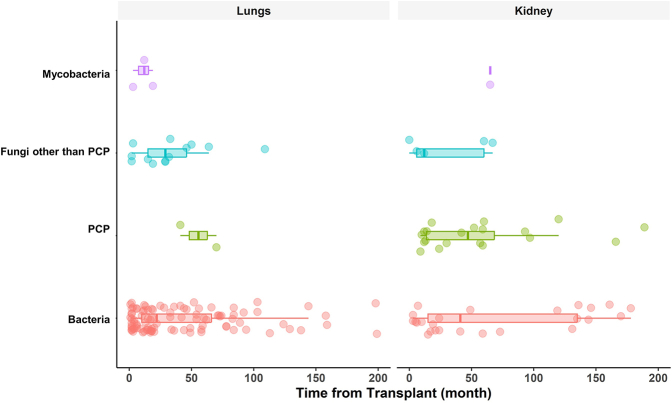


**e-Online Data:**
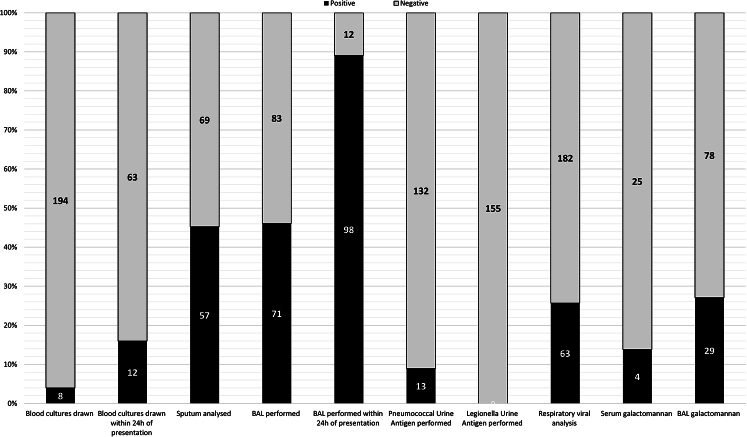


## Supplementary Data

e-Online Data

e-Online Data
