# Supplementary material for: Clinical Features, Diagnostics, Etiology, and Outcomes of Hospitalized Solid Organ Recipients With Community-Acquired Pneumonia: A Retrospective Cohort Analysis
Source: Chest. 2024 May 30;166(4):697–707. doi: 10.1016/j.chest.2024.05.005 (PMC11492221; doi:10.1016/j.chest.2024.05.005)
Supplement: e-Online Data [file mmc1.docx]

**Supplementary material**

**ICD-10 codes used to select patients**

| **ICD-10 Code** | **Label** |
| --- | --- |
| J10 | Flu, with seasonal *Influenza virus* identified |
| J11 | Flu, unidentified virus |
| J12 | Viral Pneumonia, not classified elsewhere |
| J13 | Pneumonia due to *Streptococcus pneumoniae* |
| J14 | *Haemophilus Influenzae* pneumonia |
| J15 | Bacterial pneumonia, not classified elsewhere |
| J16 | Pneumonia due to other infectious micro-organisms, not classified elsewhere |
| J17 | Pneumonia during diseases classified elsewhere |
| J18 | Pneumonia with unspecified microorganism |
| Z94.0 | Status post kidney transplant |
| Z94.1 | Status post heart transplant |
| Z94.2 | Status post lungtransplant |
| Z94.3 | Status post heart and lung transplant |
| Z94.4 | Status post liver transplant |
| Z94.6 | Status post stem cell transplant |
| Z94.8 | Status post organ transplant, not otherwise classified |

**Variables and measurement**

Definitions of excluded stays

Healthcare-associated pneumonia was defined as pneumonia in patients hospitalised within the past 90 days of onset of symptoms. Patients with hospital stays with a more likely infectious case (cholangitis, urinary tract infections, infectious diarrhoea) were also excluded. Community-acquired pneumonia stays in persons with a non-functional renal graft at the time of presentation and who weren’t on immunosuppressive medication anymore were not analysed. Coding errors included coding a history of pneumonia or redundant stays (> 1 entry in the database). Pulmonary oedema corresponds to an episode of acute heart failure without concomitant fulfilled diagnosis criteria for CAP.

Chronic lung allograft dysfunction in lung recipients was defined as a drop in the forced expiratory volume in 1 second (FEV1) of >20% from the baseline FEV1. The baseline FEV1 was defined as the average of the two highest measurements obtained at least 3 weeks post-transplant. This drop needed to persist for at least 3 weeks without any other cause ^1,2^. Acute lung allograft rejection was defined according to published histopathological criteria on transbronchial biopsy ^3^. Post-stenotic pneumonia occurred in patients with bronchial stenosis requiring bronchoscopic desobliteration. These hospitalisations were excluded due to the high risk of bias due to confounding.

We also decided not to include COVID-19 pneumonia cases because of low numbers unlikely to bring new insights in addition to the already published literature.

Data collection

All variables were collected from the medical records (transplantation reports when available, hospital stay reports, paper-based files, computerised files, radiology and laboratory records) for each patient and stay.

To describe the patient characteristics, we collected transplanted organ(s) and sex.

For each stay, we documented the time since transplantation or since first transplantation in the case of multiple asynchronous transplantations as a surrogate for begin of immunosuppressive therapy, clinical and biological signs at admission, immunosuppressive regimen, prophylaxis and previous antibiotic treatments at admission, microbiological tests and results thereof, antimicrobial therapies and their length.

The following comorbidities were documented: diabetes, a cardiovascular history, a history of chronic kidney failure, cancer still under treatment, chronic obstructive lung disease (COPD) and central nervous system (CNS) diseases that might increase the risk of aspiration.

A history of cardiovascular disease was defined as a history of coronary artery disease and/or documented heart failure (diastolic or systolic). Chronic kidney injury was defined based on average glomerular filtration rates of the past 3 months when available or documented diagnosis. COPD was not reported in patients who had received a lung transplant due to end-stage respiratory disease as a result of COPD.

Risk factors for aspiration were considered: epilepsy, a history of stroke with consequent dysphagia, and dementia.

Acute kidney injury is defined using the creatinine level at admission according to the KDIGO definition ^4^. Reduction of immunosuppressive therapy was documented when at least one immunosuppressive medication was discontinued.

Clinical course was described by respiratory insufficiency at admission or developed during stay, need for admission to an intensive care unit, need for respiratory or circulatory support, mortality during hospitalisation, at 30 days and 1 year, and length of stay. Moreover, cardiovascular complications were assessed at admission, during hospitalisation and at 1-year post-hospitalisation. These were defined as new or worsening heart failure, new atrial fibrillation or -flatter, (N)STEMI, and cardiovascular death.

Pathogen identification

An etiological diagnosis was made in the following situations: if a recognised, gram-positive, or gram-negative pathogenic bacterium was present in blood culture, sputum culture, bronchial washing, or broncho-alveolar lavage. Blood culture positivity could not be explained by an infection at another site (e.g., *S. aureus* due to endocarditis).

Respiratory viruses (coronavirus, human metapneumovirus, human rhinovirus, influenza, parainfluenza, or respiratory syncytial virus) were identified via PCR on a trans-nasal swab, bronchial washing, or BAL.

Cytomegalovirus, herpes simplex virus and varicella zoster virus were considered lung pathogens when identified by a BAL PCR, when a decision to treat was made, when other pathologies had been excluded and if the imaging was consistent with the diagnosis.

A diagnosis of invasive fungal infection was made considering the following criteria: clinical and bronchoscopic, imaging, microbiologic (positive BAL culture, serum and or BAL galactomannan, *Pneumocystis jirovecii* PCR or direct examination from the BAL), clinical decision to treat, mould or *P. jirovecii* prophylaxis at admission.

Antibiotic definitions

Nephrotoxic antibiotics: Aminoglycosides, Colistin, Teicoplanin, Vancomycin

Pseudomonas active antibiotics: Ceftaroline, Ceftazidime, Ciprofloxacin, Colistin, Levofloxacin, Piperacillin/Tazobactam, Meropenem

ORSA active antibiotics: Ceftobiprole, Linezolid, Fosfomycin, Teicoplanin, Vancomycin

References

1. Verleden GM, Glanville AR, Lease ED, et al. Chronic lung allograft dysfunction: Definition, diagnostic criteria, and approaches to treatment―A consensus report from the Pulmonary Council of the ISHLT. *J Heart Lung Transplant*. 2019;38(5):493-503. doi:10.1016/j.healun.2019.03.009

2. Verleden SE, Vos R, Vanaudenaerde BM, Verleden GM. Chronic lung allograft dysfunction phenotypes and treatment. *J Thorac Dis*. 2017;9(8):2650-2659. doi:10.21037/jtd.2017.07.81

3. Stewart S, Fishbein MC, Snell GI, et al. Revision of the 1996 Working Formulation for the Standardization of Nomenclature in the Diagnosis of Lung Rejection. *J Heart Lung Transplant*. 2007;26(12):1229-1242. doi:10.1016/j.healun.2007.10.017

4. Khwaja A. KDIGO Clinical Practice Guidelines for Acute Kidney Injury. *Nephron Clin Pract*. 2012;120(4):c179-c184. doi:10.1159/000339789
