# Supplementary material for: Clinical Features, Diagnostics, Etiology, and Outcomes of Hospitalized Solid Organ Recipients With Community-Acquired Pneumonia: A Retrospective Cohort Analysis
Source: Chest. 2024 May 30;166(4):697–707. doi: 10.1016/j.chest.2024.05.005 (PMC11492221; doi:10.1016/j.chest.2024.05.005)
Supplement: e-Online Data [file mmc2.docx]

**e-Table 1:** Detailed list of patients with single-organ or multiple-organ transplantation

|  | Patients with lung transplant and combinations of lung and other organs* | Patients with kidney transplant and combinations of kidney and other organs (exept lung)* | Patients with liver transplant* |
| --- | --- | --- | --- |
| Lung | 143 |  |  |
| Lung and heart | 12 |  |  |
| Lung and liver | 4 |  |  |
| Lung and kidney | 1 |  |  |
| Kidney |  | 121 |  |
| Kidney and pancreas |  | 15 |  |
| Kidney and liver |  | 6 |  |
| Liver |  |  | 31 |
| Total | 160 | 142 | 31 |

***** For analysis purposes, we grouped lung recipients and lung and other organ recipients (e.g., liver, kidney, heart) because the immunosuppression level is dictated by the higher levels required to prevent acute lung graft rejection. We hypothesised that the higher immunosuppression level of the patients mentioned above and the susceptibility to infection of the lung graft are substantial risk factors for CAP. Out of the remaining patients, we grouped kidney graft recipients and recipients of a kidney graft and additional organs (e.g., pancreas, heart, liver) due to similarities in immunosuppression targets and comorbidities.

**e-Table 2**: Multivariate analysis on the factors leading to an etiological diagnosis in solid-organ transplant recipients with community acquired pneumonia

| Variable | Univariate  OR (95% CI) | p-value | Multivariate  OR (%95 CI) | p-value |
| --- | --- | --- | --- | --- |
| Blood cultures performed | 1.16 (0.75 – 1.78) | 0.493 |  |  |
| Blood cultures performed within 24 h from admission | 3.75 (2 – 6.97) | <0.001 | 2.73 (1.42 – 5.25) | 0.003 |
| Sputum analysed | 0.87 (0.57 – 1.34) | 0.551 |  |  |
| BAL performed | 3.34 (2.18 – 5.13) | <0.001 |  |  |
| BAL performed within 24h from admission | 7.2 (4-13.2) | <0.001 | 6.23 (3.37 – 11.52) | <0.001 |
| Respiratory viruses assessed | 2 (1.33 – 3) | <0.001 |  |  |
| BAL galactomannan analysed | 1.5 (0.95- 2.38) | 0.084 |  |  |
| Antibiotic therapy prior to admission | 1.24 (0.8 – 1.92) | 0.335 |  |  |

**Legend**: BAL- bronchoalveolar lavage; OR – odds ratio; CI – confidence interval

**e-Table 3:** Multivariate analysis on the risk factors for a pneumonia with

*Pseudomonas aeruginosa*

| Variable | Univariate  OR (%95 CI) | p-value | Multivariate OR (%95 CI) | p-value |
| --- | --- | --- | --- | --- |
| Kidney transplant | 0.12 (0.05 – 0.3) | <0.001 |  |  |
| Liver transplant | 0.22 (0.05 – 0.92) | 0.04 |  |  |
| Lung transplant without prior chronic infection with *P. aeruginosa* | 0.45 (0.35 – 0.91) | 0.006 | 4 (1.36 – 11.8) | 0.04 |
| Lung transplant without prior chronic infection with *P. aeruginosa* | 22.07 (11.36 – 42.88) | <0.001 | 45.6 (16.98 – 122.5) | <0.001 |
| Acute graft rejection in the last 6 months | 0.99 (0.53 – 1.8) | 0.97 |  |  |
| Antibiotic treatment before admission | 1.42 (0.8 – 2.52) | 0.231 |  |  |

**Legend:** OR - odds ratio; CI – confidence interval

**e-Table 4:** Antibiotics before admission

| Variable | All stays  N = 403 | Lung Transplant Recipients  N = 198 | Liver Transplant Recipients  N = 38 | Kidney Transplant Recipients  N = 167 |
| --- | --- | --- | --- | --- |
| Antibiotics before admission – n (%) | 121 (30.1) | 66 (33.3) | 8 (21.1) | 47 (28.3) |
| Ciprofloxacin – n (%) | 42 (10,4) | 27 (13,6) | 2 (5,3) | 13 (7,8) |
| ß-lactams - n (%) | 28 (6.9) | 19 (9,6) | 4 (10,5) | 15 (8.9) |
| Oral cephalosporins – n (%) | 25 (6.2) | 7 (3,5) | 2 (5,3) | 16 (9,6) |
| Clarithromycin – n (%) | 14 (3.5) | 13 (6,6) | - | 1 (0,6) |
| Meropenem – n (%) | 5 (1,2) | 1 (0,5) | - | 4 (2,4) |
| Doxycyclin – n (%) | 3 (0,7) | 2 (1,0) | - | 1 (0,6) |
| Vancomycin – n (%) | 2 (0,5) | 1 (0,5) | - | 1 (0,6) |
| Daptomycin – n (%) | 1 (0,2) | 1 (0,5) | - | - |
